# Challenges and Approaches to Crop Improvement Through C3-to-C4 Engineering

**DOI:** 10.3389/fpls.2021.715391

**Published:** 2021-09-14

**Authors:** Hongchang Cui

**Affiliations:** ^1^Department of Biological Science, Florida State University, Tallahassee, FL, United States; ^2^College of Life Science, Northwest Science University of Agriculture and Forestry, Yangling, China

**Keywords:** C3, C4, C3-C4 engineering, gene regulatory network, systems approach, crop improvement

## Abstract

With a rapidly growing world population and dwindling natural resources, we are now facing the enormous challenge of increasing crop yields while simultaneously improving the efficiency of resource utilization. Introduction of C4 photosynthesis into C3 crops is widely accepted as a key strategy to meet this challenge because C4 plants are more efficient than C3 plants in photosynthesis and resource usage, particularly in hot climates, where the potential for productivity is high. Lending support to the feasibility of this C3-to-C4 engineering, evidence indicates that C4 photosynthesis has evolved from C3 photosynthesis in multiple lineages. Nevertheless, C3-to-C4 engineering is not an easy task, as several features essential to C4 photosynthesis must be introduced into C3 plants. One such feature is the spatial separation of the two phases of photosynthesis (CO_2_ fixation and carbohydrate synthesis) into the mesophyll and bundle sheath cells, respectively. Another feature is the Kranz anatomy, characterized by a close association between the mesophyll and bundle sheath (BS) cells (1:1 ratio). These anatomical features, along with a C4-specific carbon fixation enzyme (PEPC), form a CO_2_-concentration mechanism that ensures a high photosynthetic efficiency. Much effort has been taken in the past to introduce the C4 mechanism into C3 plants, but none of these attempts has met with success, which is in my opinion due to a lack of system-level understanding and manipulation of the C3 and C4 pathways. As a prerequisite for the C3-to-C4 engineering, I propose that not only the mechanisms that control the Kranz anatomy and cell-type-specific expression in C3 and C4 plants must be elucidated, but also a good understanding of the gene regulatory network underlying C3 and C4 photosynthesis must be achieved. In this review, I first describe the past and current efforts to increase photosynthetic efficiency in C3 plants and their limitations; I then discuss a systems approach to tackling down this challenge, some practical issues, and recent technical innovations that would help us to solve these problems.

## Introduction

About half a century ago, billions of people were on the brink of starvation. Fortunately, this potential disaster was avoided, thanks to the Green Revolution, which dramatically improved crop yields by introducing crops of higher productivity as well as new measures and materials for crop management. Since then, the Green Revolution has been a major driving force for an agriculture that has so far met the need of a rapidly growing world population. Another worldwide food crisis is looming, however, as increase in crop yield is reaching a plateau while the world population is still growing rapidly. According to a recent report from the United Nations, by 2050 the population is estimated to be 9.7 billion, an increase of 2.7 billion beyond the present population (World Population Prospects: the 2019 Revision).^1^ To meet the demand for food, world food production will need to increase by 70%. With the current crops, this means the use of more land, water, fertilizers, pesticides, and herbicides, which are not only energy expensive but also detrimental to the environment. A sustainable agriculture calls for a second Green Revolution, whereby crops with higher productivity and better efficiency in resource use must be developed (Wollenweber et al., 2005).

Crop productivity is determined by a number of factors, such as the percentage of sunlight interception, photosynthetic efficiency, and the proportion of resources allocated to seeds (Foley et al., 2011). By increasing crop density combined with fertilizer utilization and irrigation, the percentage of sunlight interception has been maximized during the first Green revolution (de Bossoreille de Ribou et al., 2013). The proportion of resources allocated to seeds has also been increased dramatically through breeding for cultivars with a short stature and large seeds (Long et al., 2015). The major factor that still needs to be improved is photosynthetic efficiency. It is estimated that photosynthetic efficiency is less than 4.6% in C3 plants, whereas in C4 plants this number can reach 6% (Zhu et al., 2008). Hence, there is a great potential to increase photosynthetic efficiency and thus productivity.

Depending on the structure of the first product of the CO_2_ fixation reaction, plants can be classified into two major groups: C3 plants, which make a three-carbon compound, 3-phosphoglycerate, or C4 plants in which a four-carbon product, oxaloacetate (OAA), is synthesized (Gowik et al., 2011). C4 photosynthesis is more efficient than C3 photosynthesis in warmer climates, where yield potential is high. The lower photosynthetic efficiency in C3 plants is due to a dual activity in the enzyme that fixes CO_2_, Ribulose-1,5-bisphosphate carboxylase/oxygenase (RuBisCO; Brown et al., 2011). Besides its carboxylase activity, RuBisCO also has an oxygenase activity, which turns Ribulose-1,5-bisphosphate (RuBP), the substrate for CO_2_ fixation, into 2-phosphoglycolate. To regenerate RuBP from 2-phosphoglycolate, ATP is consumed and CO_2_ is released, leading to loss of carbon and reduction in photosynthetic efficiency. Because this process resembles respiration, it has been named photorespiration (Sage et al., 2012). The oxygenase activity of RuBisCO is enhanced under intense light and high temperature, which is why C3 plants are less competitive than C4 plants in tropical and subtropical climates. C4 plants do not have this issue because they rely on a different enzyme for CO_2_ fixation, PEP carboxylase (PEPC), which does not have oxygenase activity (Gowik et al., 2011). Many important crops are C3 plants, such as rice, wheat, and soybean. Introducing the C4 mechanism into these crops provides an attractive means to meet the need of a rapidly growing world population (von Caemmerer et al., 2012; Long et al., 2015). It is estimated that an increase of as little as 10% in photosynthetic efficiency would increase crop yields by 50% (Langdale, 2011).

C4 plants are also more efficient in nitrogen and water use. Owing to its low efficiency, RuBisCO is produced in large amount in C3 plants. As a matter of fact, RuBisCO is the most abundant protein in the world, accounting for nearly 50% of the total proteins in leaves. Because PEPC has a high affinity for CO_2_, C4 plants can fix CO_2_ at a much lower level of atmosphere CO_2_ and can maintain photosynthesis even when the stomata are not completely open, thus reducing water loss.

Evidence suggests that C4 plants have evolved from C3 plants in more than 60 distinct lineages (Christin et al., 2013). There are thousands of plant species using the C4 photosynthesis, including both dicots and monocots. The large number of C4 plants and independent events of C4 evolution suggest that the C3-to-C4 conversion is a relatively easy step in evolution. Importantly, these findings also lend support to the feasibility of the C3-to-C4 engineering, which has drawn enormous interests (Hibberd et al., 2008; Furbank, 2017; Sedelnikova et al., 2018). Nevertheless, C3-to-C4 engineering still poses an enormous challenge, as discussed below.

## Text

### C4 Photosynthesis Is a Syndrome

In addition to the deployment of an oxygen-insensitive carboxylase (PEPC), C4 plants have acquired a number of features that collectively improve their photosynthetic efficiency (Sage et al., 2012). One of the most critical features is the spatial separation of the two phases of photosynthetic process, i.e., CO_2_ fixation and the Calvin-Benson cycle, into mesophyll and BS cells. Thus, BS cells become the primary sites of photosynthesis, whereas mesophyll cells are only involved in CO_2_ fixation. In C3 plants, in comparison, photosynthesis occurs in both mesophyll and BS cells, although in many cases BS cells contribute little to photosynthesis (Kangasjarvi et al., 2009). In C4 plants, the BS cells are also much larger and contain a greater number of chloroplasts that are also enlarged. On the other hand, RuBisCO expression is dramatically reduced or completely absent in mesophyll cells, thus minimizing carbon loss in the mesophyll cells. The spatial separation of the photosynthetic process thus forms a CO_2_ concentration mechanism (Hatch and Osmond, 1976). Photorespiration in the BS cells is suppressed due to a high concentration of CO_2_. The internal position of the BS cells in leaves makes them less accessible to O_2_ in the atmosphere, resulting in an increase in the ratio between CO_2_ and O_2_ and consequently further suppression of photorespiration.

Compared to C3 plants, C4 plants also have a more active and dynamic system for metabolite transport between mesophyll and BS cells (Weber and von Caemmerer, 2010; Gowik et al., 2011). An efficient metabolite transport system is essential to C4 photosynthesis because on the one hand, photosynthesis in BS cells relies on a continuous supply of CO_2_ (in the form of a C4 compound) from the mesophyll cells, and on the other hand, the substrate for CO_2_ fixation is regenerated in the BS cells through decarboxylation of the C4 compound, and this compound has to be shuttled back into the mesophyll cells. Removal of the C4 compound from the mesophyll cells and the decarboxylation product from the BS cells is also necessary because their accumulation would inhibit CO_2_ fixation and the decarboxylation reaction, respectively.

**Figure 1:**
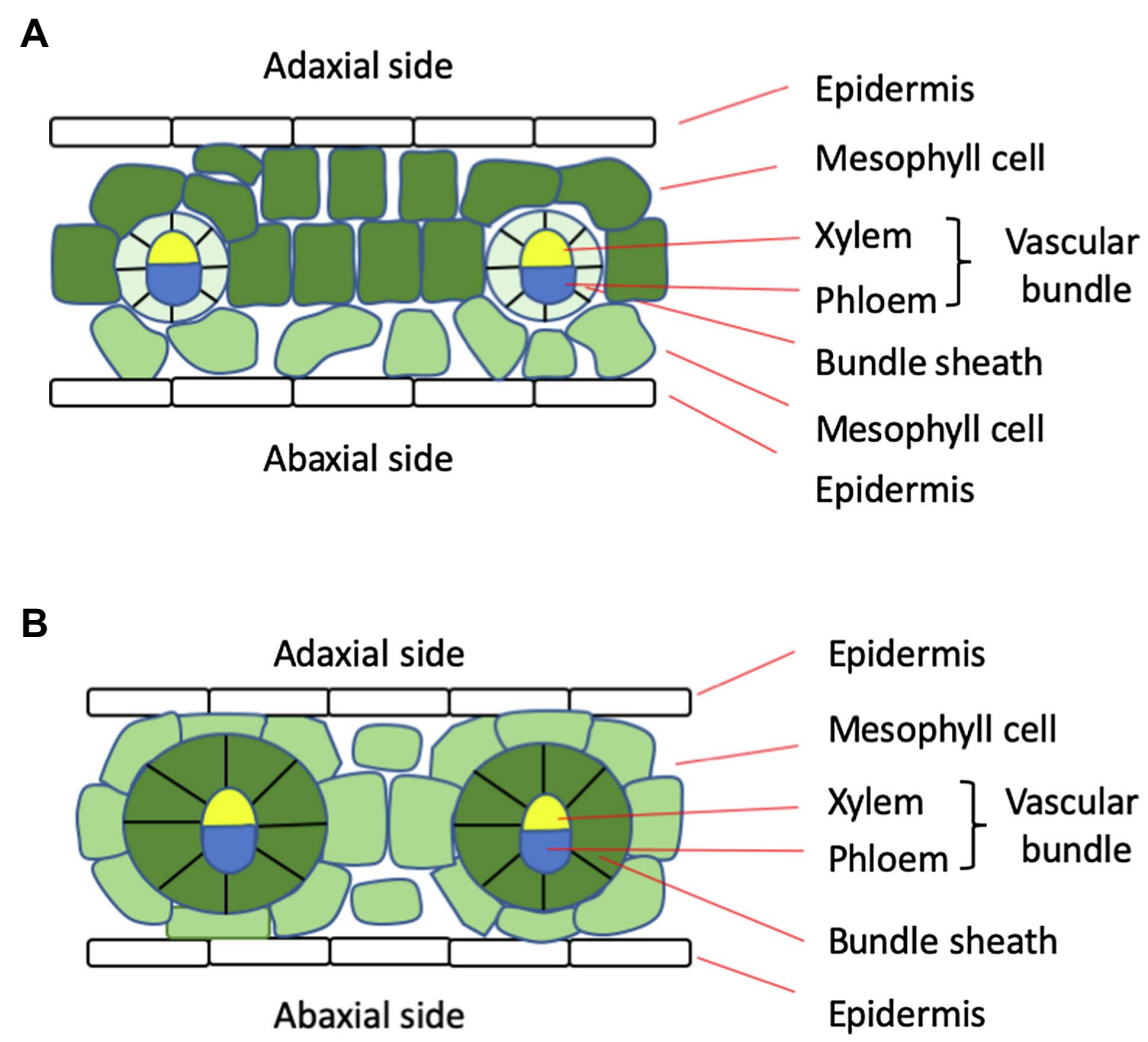
Diagram of the leaf anatomy of typical C3 **(A)** and C4 **(B)** plants. In C4 plants, there are two mesophyll cells between neighboring vascular bundles, whereas in C3 plants, there are more than two. Also, the bundle sheath cells in C4 plants are much larger and contain more chloroplasts, as indicated by color intensity.

Another important feature of C4 plants is the Kranz anatomy, characterized by a 1:1 ratio between the BS cells and mesophyll cells, in contrast to a ratio greater than 2 in C3 plants (Figure 1; Wang et al., 2011). Along with higher expression levels of transporters and a large number of plasmodesmata in the cell wall between mesophyll and BS cells (Danila et al., 2016), the Kranz anatomy facilitates metabolite exchange between the two cell types. Because these cells form centric rings surrounding the vascular tissue, transport of nutrients and water from the vascular tissue is also more efficient.

Not all C4 plants use the two-cell CO_2_ concentration mechanism, though. In some C4 plants, the whole photosynthetic process is completed within the same cell (Voznesenskaya et al., 2001). Plants with Crassulacean acid metabolism (CAM), which can be considered a special type of C4 photosynthesis, also perform photosynthesis within the same cell (Black and Osmond, 2003). In the former case, increased photosynthetic efficiency is achieved through spatial separation of the two phases of photosynthesis into different parts of the cell. In CAM plants, however, this is achieved through temporal separation, with CO_2_ fixation and the Calvin-Benson cycle taking place in the night and day, respectively. This one-cell mechanism thus enables plants to conduct photosynthesis in a dry and hot climate because stomata close in the day, avoiding water loss, but open in the night, allowing gas exchange and CO_2_ fixation (DePaoli et al., 2014).

### C3-to-C4 Engineering: One Cell or Two Cell?

Some genera, such as Flaveria and Cleome, contain both C3 and C4 species (Brown et al., 2005; Marshall et al., 2007). Because interspecies hybrids can be produced from some of these species, these genera provide valuable resources for early attempts of C3-to-C4 engineering as well as studies of the mechanisms of C4 photosynthesis (Brown and Bouton, 1993). Although some F1 hybrids show a higher rate of photosynthetic efficiency, increased vein density, and even a higher yield, most resemble their C3 parents (Brown and Bouton, 1993). Even in hybrids with improved photosynthesis, the hybrid vigor becomes dissipated in subsequent generations due to segregation of the multiple loci-controlled C4 trait (Brown and Bouton, 1993). Another issue is that most F1 hybrids are sterile, making it impossible to maintain the germplasm.

A major limitation of this genetic-crossing-based method is that it can only be used to C3 species with closely related C4 relatives. Unfortunately, most of the world’s important crops do not have a close C4 relative. A solution to this interspecies barrier is protoplast fusion (Bates, 1985), which has been used to generate C3-C4 hybrids (Mastuti et al., 1997). Protoplast fusion, however, does not solve the problem of sterility and instability of the C4 trait, which has hindered its application in C3-to-C4 engineering.

Because of the complexity of the two-cell C4 mechanism, subsequent efforts of C3-to-C4 engineering have therefore focused on the one-cell mechanism. As the first step in this C3-to-C4 engineering, PEPC was overexpressed in both mesophyll and BS cells. The rationale for this one-cell approach is that PEPC expression should reduce photorespiration and thereby increase the photosynthetic efficiency and yield. Although photosynthetic efficiency was increased in the resulting transgenic plants, plant growth and yield were not improved (Miyao et al., 2011) and in some cases, there was even a drop in the yield (Taniguchi et al., 2008). Similarly, increase in yield has not been achieved when other genes essential to C4 photosynthesis were overexpressed in both mesophyll and BS cells (Ruan et al., 2012).

CO_2_ concentration mechanisms have been found in other organisms. In cyanobacteria, for example, an elevated level of CO_2_ is maintained around RuBisCO by a structure called carboxysome, which forms a shell around RuBisCO and carbonic anhydrase (Rae et al., 2013). Compared to the CO_2_ concentration mechanism in C4 plants, this mechanism is much less complex and hence could be more suitable to C3-to-C4 engineering (McGrath and Long, 2014). This idea was tested by transgenic studies (Lin et al., 2014). Surprisingly, although the photosynthetic efficiency was improved, plant growth was severely affected (Lin et al., 2014). These studies suggest that, to convert a C3 plant into a C4 plant, not only a CO_2_ concentration mechanism but also other features that make photosynthesis more efficient must be introduced into C3 plants. The two-cell approach has gained favor in current C3-to-C4 engineering efforts, as plants with two-cell C4 photosynthesis are generally more productive than plants with the CAM mechanism.

### Engineering a Two-Cell C4 Photosynthesis

To engineer a two-cell C4 mechanism, the following modifications should be made in C3 plants: (1) Expression of genes involved in C4 photosynthesis in a cell-type-specific manner. (2) Increase in the number of vascular bundles. (3) Enlargement of BS cells. (4) Increase in the number of chloroplasts in each BS cell. (5) Enhancement in the metabolite shuttling system between mesophyll and BS cells. To modify these features, we need to have a good understanding of the mechanisms that determine cell-type-specific gene expression, chloroplast development, and the Kranz anatomy.

#### Cell-Type-Specific Gene Expression

Cell-type-specific expression is critical to C4 photosynthesis, as it is part of the mechanism underlying the spatial separation of the photosynthetic process. First, mesophyll-cell-specific expression of PEPC, along with low RuBisCO expression in this cell type, ensures efficient CO_2_ fixation. Second, 2-oxyglutarate/malte transporter (OMT), a plastid-membrane-localized transporter in mesophyll cells, is needed to transport OAA, the primary C4 compound generated in the CO_2_ fixation reaction, from the cytoplasm into the chloroplast. NADP malate dehydrogenase (NADP-MDH) then converts OAA into malate, which is exported into the BS cells by dicarboxylate transporter (DIT1). In the BS, malate is decarboxylated by NADP-ME, releasing pyruvate and CO_2_. While CO_2_ is used in the Calvin cycle for carbohydrate biosynthesis, pyruvate is transported by proton/pyruvate symporters (MEP) back into the mesophyll cells where it is converted into phosphoenolpyruvate by pyruvate Pi dikinase (PPDK), thus regenerating the substrate for CO_2_ fixation.

In C4 plants, PEPC, OMT, NADP-MDH, PPDK, and carbonic anhydrase (CA) are expressed specifically in mesophyll cells, whereas RuBisCO, DIT1, and NADP-ME are expressed preferentially in the BS (Weber and von Caemmerer, 2010; Gowik et al., 2011). Most of these genes are expressed in C3 plants as well but in a non-cell-type-specific manner. To engineer a two-cell C4 mechanism in C3 plants, therefore, the expression pattern of these genes must be modified. For this purpose, mesophyll- and bundle-sheath-cell-specific promoters are required. Such cell-type-specific promoters are also needed to express other genes essential to C4 photosynthesis. To identify promoter sequences that can be used for C3-to-C4 engineering, the expression patterns of some C4 cell-type-specific genes in C3 plants have been examined. Surprisingly, most cell-type-specific promoter sequences examined so far are unable to maintain their cell-type expression pattern in C3 plants (Hibberd and Covshoff, 2010; Reeves et al., 2017). The PEPC promoter from maize (Matsuoka et al., 1994) and the GLDPA promoter from *Flaveria trinervia* (Engelmann et al., 2008; Wiludda et al., 2012) are among the very few promoters that confer cell-type-specific expression in C3 plants. A common problem is that BS-specific promoters expand their expression into the vascular tissue, whereas mesophyll-cell-specific promoters become expressed in both mesophyll and BS cells (Schaffner and Sheen, 1991; Stockhaus et al., 1994; Nomura et al., 2005; Akyildiz et al., 2007). More robust mesophyll- or BS cell-specific regulatory sequences of different strength are needed.

The distinct expression patterns of the same regulatory sequences in C3 and C4 plants are believed to be due partly to the difference in gene regulatory mechanisms between C3 and C4 plants (Matsuoka et al., 1994; Nomura et al., 2000), and partly to the different cellular environmental conditions, such as sugar content, redox, and other metabolites, which appear to have a large effect on gene expression (Schaaf et al., 1995; Covshoff et al., 2008; Brown et al., 2011). There is also evidence for the involvement of posttranscriptional (Wiludda et al., 2012) and epigenetic regulation (Ngernprasirtsiri et al., 1989; Tolley et al., 2012). Another explanation is that not all the necessary regulatory elements in a particular promoter have been used for exogenous expression. It is a common practice to take the sequence upstream of the coding region as the promoter, but in many genes the 5'untranslated region, the first intron, or even the 3'-untranslated region have important regulatory roles (Chung et al., 2006; Patel et al., 2006; Schauer et al., 2009; Kajala et al., 2012). In *Cleome gynandra*, an emerging C4 model plant (Brown et al., 2005), a 240-bp fragment in the translated region of both NAD1 and NAD2 has also been shown to be essential for BS-specific expression (Brown et al., 2011). Subsequent studies showed that some mesophyll-specific genes are also regulated by exons (Williams et al., 2016) and that exons with a dual role in protein coding and gene regulation are widespread among C4 plants (Reyna-Llorens et al., 2018).

More recently, a bipartite transcription factor module controlling expression in the BS of *Arabidopsis thaliana* was reported (Dickinson et al., 2020). Based on this finding, a short and tunable promoter was synthesized, which is able to confer BS-specific expression (Dickinson et al., 2020). Such synthetic promoters are desired in C3-to-C4 engineering as they can greatly reduce the size of transgenes and thereby increase the efficiency of transgene expression.

#### The Kranz Anatomy

Because of the crucial role of the Kranz anatomy in C4 photosynthesis, extensive efforts have been exerted to elucidate the mechanisms that control this anatomical feature (Sedelnikova et al., 2018). To this end, a series of experiments have been conducted with the grass *Alloteropsis semialata*, which has emerged as an excellent model plant to study C4 evolution because it includes all photosynthetic types ranging from C3 to C3-C4 intermediates and C4 (Lundgren et al., 2015, 2016; Dunning et al., 2017). Strikingly, vein density was found to be the only feature that distinguish these photosynthetic types (Lundgren et al., 2016). This result suggests that vein intensity is a major factor driving C3-to-C4 transition (Lundgren et al., 2016), which is conceivable as increase in vein density would reduce the ratio between mesophyll and BS cell layers. Using genetic screens, a number of mutants defective in vascular patterning have been identified in *Arabidopsis thaliana* (Petricka et al., 2008; Huang et al., 2017) and sorghum (Rizal et al., 2015). However, the vein patterning defects in these mutants are attributed to mutations in factors involved in hormone biosynthesis or signaling, which are difficult to control and thus have little value in C3-to-C4 engineering.

Transcription factors play critical roles in development. Thus, to modify leaf anatomy requires knowledge of transcription factors that control the Kranz anatomy. To identify such factors, a comparative transcriptome approach has been used in some studies. For example, two independent groups have examined the genes that are expressed in different parts of a young maize leaf, which represent different developmental stages (Li et al., 2010; Wang et al., 2013), or in different organs with (foliar leaf blade) or without (husk leaf sheath) the Kranz anatomy (Wang et al., 2014). A number of genes associated with the Kranz anatomy were identified, although their function still remains largely uncharacterized. Transcriptome analysis has also been conducted with *Alloteropsis semialata*, which uncovered a small number of genes differentially expressed in C3, C3-C4 intermediates, and C4 plants (Lundgren et al., 2019). However, these genes all code for enzymes, which are of low value in C3-C4 engineering.

BS cells are critical to C4 photosynthesis but studies about their developmental pathways are scarce. To identify BS cell fate determinants, a genetic screen has been performed with fox millet (Luo et al., 2018). A number of mutants with abnormal leaf anatomy were recovered in this screen (Luo et al., 2018), but the genes with the causal mutations have yet to be located. When misexpressed, the transcription factors NAC052 caused an increase in the number of BS cells (van Rooijen et al., 2020), suggesting a role in Kranz anatomy. However, NAC052 is unlikely to be a BS cell fate determinant factor because BS cells are still present in the NAC052-misexpressing plants.

A lack of understanding of the molecular mechanisms underlying BS development has been a rate limiting factor in C3-C4 engineering. Filling this critical gap of knowledge, we have demonstrated that three members in the GRAS family of transcription regulators constitute a developmental pathway regulating BS development in *Arabidopsis thaliana* (Cui et al., 2014). Anatomically, the BS is analogous to the endodermis in root, as they both form a single cell layer around the central vascular tissue. Hence, it has been suggested that factors that determine endodermis cell fate specification may also regulate BS development (Slewinski, 2013; Wang et al., 2013). Two members of the GRAS family of transcription regulators, SHORT-ROOT (SHR) and SCARECROW (SCR), are known to control endodermis specification in the Arabidopsis root (Di Laurenzio et al., 1996; Helariutta et al., 2000); we have therefore examined the leaf anatomy in *shr* and *scr* mutants (Cui et al., 2014). In the wild type, BS cells can be easily discerned from the mesophyll cells by their small size and rectangular shape. In the *shr* mutant, cells surrounding the vascular tissue are enlarged and irregular in shape, which are characteristics of mesophyll cells. In the *scr* mutant, however, the BS cell layer is still present, although its cell size is somewhat larger. This observation suggests that SCR plays a role in BS development, but other factors are also involved. In further studies, we found that SCL23, a close homolog of SCR (Bolle, 2004), is also required for BS development.

**Figure 2:**
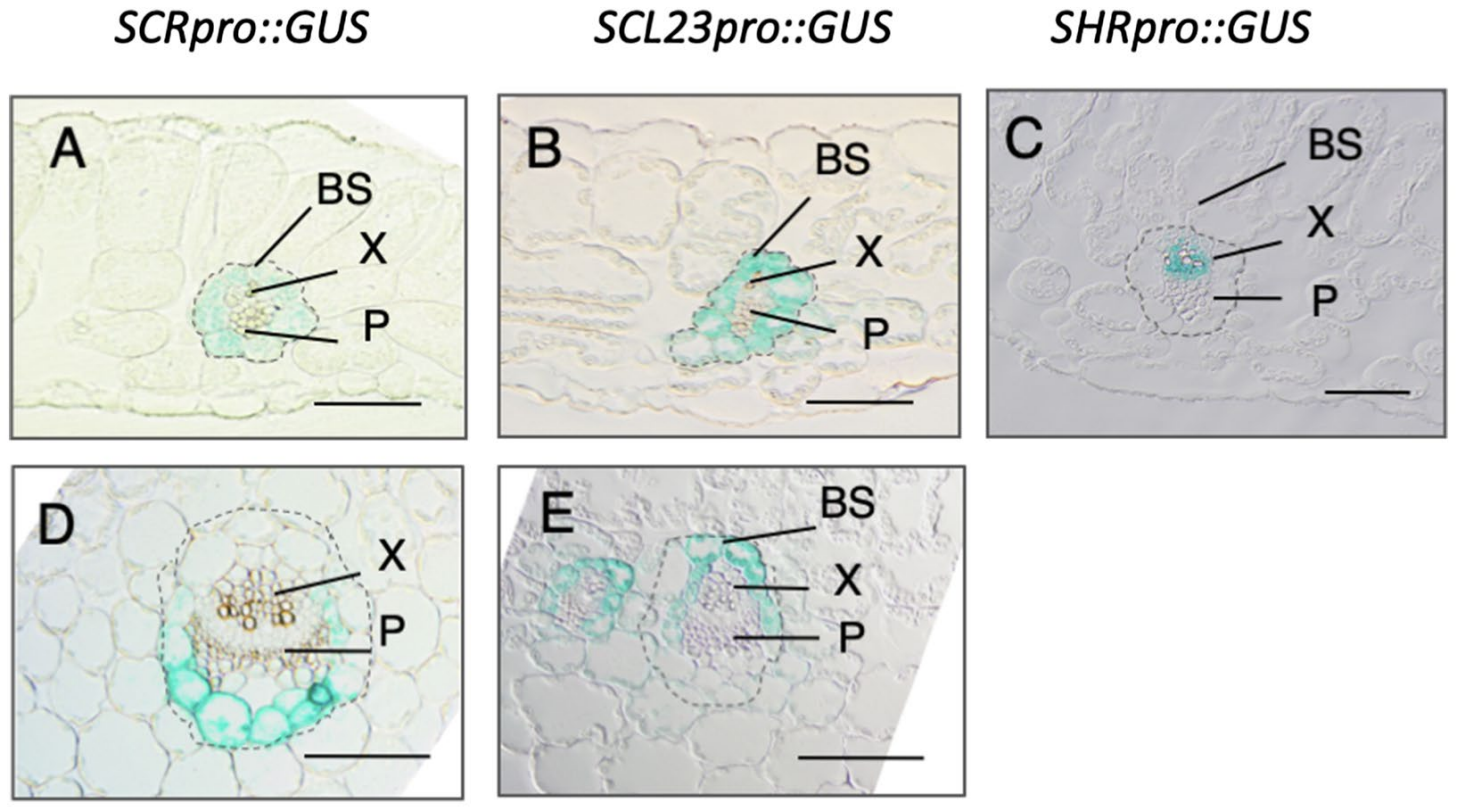
Cross section of Arabidopsis leaves showing the expression pattern of SCR, SCL23, and SHR revealed by GUS staining. In young leaves, SCR and SCL23 are expressed in all bundle sheath (BS) cells but in mature leaves, they become restricted to the BS cells associated with the phloem (P) and xylem (X), respectively. **(A-C)**. Young leaves; **(D)** and **(E)**. Old leaves. (Adapted from Cui et al., 2014. Plant Journal. © 2014 John Wiley & Sons Ltd).

Consistent with their redundant roles in BS development, SCR and SCL23 are both expressed specifically in the BS cells (Cui et al., 2014; Figures 2A,B). In contrast, SHR is expressed in the vascular tissue (Figure 2C). However, the SHR protein is also present in the adjacent cell layer due to intercellular trafficking (Gardiner et al., 2011). SCR and SCL23 are under the control of SHR, and together, they define the BS cell fate and pattern. Notably, although SCR and SCL23 are expressed uniformly in the BS cells during early leaf development, their expression patterns become distinct at later stages of leaf development (Cui et al., 2014; Figures 2D,E). While SCR becomes preferentially expressed in the BS cells associated with the phloem at the abaxial side of the leaf, SCL23 expression is restricted to those cells that are associated with the xylem at the abaxial side of the leaf (Cui et al., 2014). The functions of the two genes also differ at later stages of leaf development, with SCR being primarily involved in sugar and amino acids transport and SCL23 playing a major role in water and mineral transport. The SHR-SCR-SCL23 developmental pathway is likely to be evolutionarily conserved, as homologs to SHR, SCR, and SCL23 are present in higher plants (Engstrom, 2011) and, in many plants examined so far, these genes have expression patterns similar to those of their Arabidopsis counterparts, at least in the roots (Lim et al., 2000, 2005; Sassa et al., 2001; Kamiya et al., 2003; Cui et al., 2007; Sbabou et al., 2010).

Contrary to the widely accepted notion that the BS comprises a single cell type, our study also reveals the existence of two types of BS cells, one associated with the xylem and the other with the phloem (Cui et al., 2014). This finding suggests that the two cell types must be manipulated separately to avoid disturbance of the physiology in the leaf (Gao et al., 2014). It also suggests that promoters that confer specific expression in these two types of BS cells should be used for engineering. In this regard, the SCR and SCL23 promoter sequences would provide invaluable tools for these needs.

SHR and SCR appear to also play a pivotal role in the Kranz anatomy. In maize, a C4 plant, mutations in the SHR or SCR homolog cause defects in cell patterning in the leaves, characterized by a variable number of mesophyll cells surrounding the BS (Slewinski et al., 2012, 2014). Surprisingly, however, these mutants still have normal BS cells, unlike the Arabidopsis *shr* and *scr* mutants. Further studies revealed that maize SCR and its homolog are not required for BS development but rather control mesophyll cell differentiation (Hughes et al., 2019). Intriguingly, the double mutant no longer has the endodermis (Hughes et al., 2019), suggesting that SCR homologs do not necessarily have the same function in different plant species. It is noteworthy, though, that three SCR homologs can be identified in the maize genome (Guo et al., 2017 and our unpublished data), thus the possibility that SCR and homologs do not affect BS development cannot be excluded yet. Further studies are needed to distinguish between these possibilities.

#### Chloroplast Size and Number

Compared to C4 plants, C3 plants have only a few or even no chloroplasts in their BS cells, largely owing to their small cell size. Thus, to engineer a C4 mechanism in C3 plants, the BS cell size must be increased. This could be achieved by manipulation of the expression level or pattern of SCR, because mutation in SCR causes enlargement of BS cells in *Arabidopsis thaliana* (Cui et al., 2014). There is also evidence that SCR is required to establish and/or maintain photosynthetic capacity in maize leaves (Hughes and Langdale, 2020). Another way to increase the number of chloroplasts in BS cells is by manipulating the activity of factors involved in chloroplast development.

Several maize genes are known for their role in chloroplast development specifically in BS cells (Kinsman and Pyke, 1998), such as GOLDEN2 and related transcription factors (Hall et al., 1998; Rossini et al., 2001), and BUNDLE SHEATH DEFECTIVE 2 (BSD2), which encodes a DnaJ-like protein that regulates *rbcL* expression in the BS cells (Brutnell et al., 1999). When a GOLDEN2-like gene from rice or the BSD2 gene from maize was overexpressed in rice or maize, chloroplast development was induced in the BS, indicating that these two genes are master regulators of chloroplast development (Wang et al., 2017). Interestingly, overexpression of the rice GOLDEN2-like gene also resulted in other modifications that would facilitate C4 photosynthesis, such as enlargement of chloroplast and mitochondria, an elevated level of photosynthetic enzymes in the BS cells, and a higher density of plasmodesmata in the cell wall between the BS and mesophyll cells (Nakamura et al., 2009; Wang et al., 2017). Notably, however, the effect of the endogenous GOLDEN2-like gene on chloroplast development was observed only in callus and young seedlings, which was attributed to gene silencing at the level of posttranscriptional or translational regulation (Wang et al., 2017). Regardless of the cause, this issue can be overcome by overexpressing a maize GOLDEN2-like gene in rice (Wang et al., 2017). Overexpression of the maize GOLDEN2-like gene also improves photosynthetic efficiency and reduces photoinhibition, thereby enhancing biomass and grain yields in rice (Li et al., 2020). These results demonstrate that GOLDEN2 is a powerful tool for C3-to-C4 engineering.

In addition to GLK and BSD2, a number of genes involved in chloroplast division have also been identified, including GNC and CGA1 (Chiang et al., 2012), GROWTH REGULATING FACTOR5 (GRF5; Vercruyssen et al., 2015), two FtsZ-like factors (Osteryoung et al., 1998), two plastid chaperonin proteins (Cpn60 alpha and Cpn60 beta; Suzuki et al., 2009), a cytosolic dynamin-like protein (ARC5; Gao et al., 2003), CPD45 (Chang et al., 2015), PARC6 (Glynn et al., 2009), and MinD (Dinkins et al., 2001), and alteration in the expression level or activity of some of these factors leads to changes in the number and size of chloroplasts. For instance, when expressed at a higher level or when the ATPase activity is reduced, the MinD gene from *Arabidopsis thaliana* causes an increase in the number of chloroplasts in tobacco (Dinkins et al., 2001). However, another consequence of these transgenes is smaller chloroplasts. These unwanted effects may be suppressed by co-expression of other genes, as the role of MinD in chloroplast development can be modified by other chloroplast division factors (Chikkala et al., 2012).

### Gene Regulatory Networks

Although many genes with an important role in C4 photosynthesis have been identified, they probably represent only a fraction of the repertoire of genes required for installing a C4 mechanism in C3 plants. Indeed, a comparative study of the transcriptome in the leaves of two closely related C3 and C4 species, *Cleome spinosa* and *Cleome gynandra*, shows that more than 600 genes are expressed at a higher level in the C4 species (Brautigam et al., 2011). Although not all of these differentially expressed genes may be associated with the C4 pathway, a considerable fraction of them are likely to be.

From an engineering perspective, it is clearly not feasible to modify the expression level of all these genes individually. Increase in the number of transgenes not only increases the time and cost, but also increases the risk of gene silencing and genome disruption. It is therefore crucial to minimize the number of transgenes. By comparing the gene regulatory networks underlying C3 and C4 photosynthesis would help us to minimize the number of genes that need to be manipulated because, according to the prevailing view, C4 mechanisms have been built on existing C3 gene networks (Brown et al., 2011; Reyna-Llorens and Hibberd, 2017). and this comparative approach would enable us to identify master regulators of C4 mechanisms. Supporting this view, there is ample evidence that C4 photosynthesis is derived from C3 plants (Christin et al., 2013) and that many genes share the same cis-regulatory elements in C3 and C4 plants (Kajala et al., 2012; John et al., 2014). C4 photosynthesis is a complex process and therefore, for optimal performance, all of its components should act coordinately. Knowledge about the C4 gene regulatory network will also enable us to integrate these individual components.

As a first step toward deciphering the C4 gene regulatory network, genome-wide studies have been conducted to define the lists of genes with a role in various features of C4 photosynthesis. For example, in order to identify genes required for the formation of the Kranz anatomy, comparative transcriptomic analyses have been performed with different regions of a young maize leaf (corresponding to different developmental stages; Wang et al., 2013) or with different organs with or without the Kranz anatomy (Wang et al., 2014). This approach not only identifies SHR and SCR as potential regulators of Kranz anatomy but also suggests that other pathways may play a role as well in the development of the Kranz anatomy, a finding that deserves further investigation. Similar studies have been performed with *Alloteropsis semialata* (Dunning et al., 2017) and *Cleome gynandra* (Aubry et al., 2014), which also has become an excellent model plant for C4 research owing to its small genome size, short stature, and fast life cycle (Newell et al., 2010). Because all *Alloteropsis semialata* plants with different photosynthetic types belong to the same species (Lundgren et al., 2015, 2016), theoretically, they should allow us to pinpoint the most critical regulators of C4 photosynthesis. Surprisingly, however, none of the Kranz anatomy regulators identified in other studies, including SHR, SCR, and SCL23, was uncovered in the study with this plant. One explanation for this observation is that Kranz anatomy regulators are already installed in C3 plants, as suggested by others (Reyna-Llorens and Hibberd, 2017), and that changes in their spatial expression patterns through rewiring the gene regulatory network, rather than changes in gene expression levels, underpin the quantum jump from C3 or C3-C4 intermediates to C4 photosynthesis. Thus, to decipher the core C4 gene regulatory network, the information from various studies should be combined and meta-analyzed.

To understand the gene regulatory network in the BS cells, whole-genome transcriptome and proteomics experiments have also been conducted with mesophyll and BS cells isolated from maize and rice leaves (Sawers et al., 2007; Nelson, 2011; Chang et al., 2012; Aubry et al., 2014; Hua et al., 2021). A large number of genes that appear to be expressed preferentially in mesophyll or BS cells have been identified. This information should not only facilitate the identification of cell-type-specific promoters but also provide important insights into the molecular basis of C4 photosynthesis. In maize, for example, genes that are involved in cell wall modification for intercellular transport appear to be expressed specifically in the BS cells (Chang et al., 2012). This finding suggests that the BS cells play a critical role in defining the cell wall features that facilitate intercellular transport. It further suggests that expressing cell wall modification genes solely in the BS cells might be sufficient to confer C4 properties.

Determination of the direct relationship between genes is key to defining the C4 gene regulatory network, but research in this direction is still very rudimentary. To our knowledge, to date our recent work on genome-wide determination of direct targets of SHR, SCR, and SCL23 in Arabidopsis leaves represents the only work in this direction (Cui et al., 2014). Cleary, future efforts should be directed to address this need.

### Molecular Tools for C3-to-C4 Engineering

To construct a two-cell C4 mechanism presents at least two critical technical challenges. The first concerns the number of genes whose expression patterns and levels need to be modified. Although this number can be dramatically reduced through manipulation of master regulators of pathways or processes, it is unlikely that all genes can be regulated by this strategy. The many features of C4 photosynthesis that need to be introduced into C3 plants mean a considerable number of transgenes must be constructed separately. Because reduction in the number of transgenes not only reduces the amount of time and cost but also reduces the problems associated with transgene silencing and genome disruption, it is of great importance to further reduce the number of transgenes. The second technical challenge is that novel tools are needed to reduce gene expression in a cell-type-specific manner. Although RNA interference has proven a powerful tool for gene knockdown, it is not suitable for this purpose because miRNA is mobile and the same target gene will be affected in the neighboring cells, in which a higher level of gene expression may be necessary.

Our ability to deal with these technical challenges has been significantly enhanced by a burst of novel gene cloning and genome editing technologies in recent years. The Golden Gate cloning method is of particular interest because it allows the stacking of multiple genes in a straightforward manner (Lee et al., 2020). Using this method, Ermakova et al. recently demonstrated that a C4 photosynthetic pathway can be installed in rice by simultaneously expressing five maize photosynthetic enzymes using a single construct (Ermakova et al., 2021). Another powerful tool is the CRISPR-Cas method, which is not only highly efficient and precise but also very versatile in gene editing (Cermak et al., 2017; Zhang et al., 2019; Zhu et al., 2020). There are two components in the CRISPR-Cas system: a guide RNA, which directs the Cas9 protein to target genes through sequence complementarity and the CRISPR-associated protein 9 (Cas 9), which is a nuclease (Horvath and Barrangou, 2010). In its initial form, the CRISPR-Cas method is used to create lesions in target genes, but it can also insert a DNA fragment into the target sequence if a DNA fragment flanked by sequences homologous to the target sites is provided. The sequence replacement function of the CRISPR-Cas method is particularly useful for C3-to-C4 engineering because once the target sequence has been modified, the transgene can be eliminated, thus avoiding some of the issues associated with transgenes, such as disruption of the genome or transgene silencing (Cong et al., 2013; Feng et al., 2014). It has been shown that some C4 genes have acquired cis-regulatory elements in their promoter sequences (Hibberd and Covshoff, 2010); these genes would be perfect targets for modification using the sequence replacement function of the CRISPR-Cas method.

The CRISPR-Cas system can also be used to increase or decrease gene expression by replacing the nuclease domain in Cas9 with transcription activation domain or repressor domain (Sander and Joung, 2014; Zhang et al., 2019). Due to its large size (160kDa), Cas9 cannot move between cells and thus allows for cell-type-specific regulation of gene expression when expressed under a cell-type-specific promoter. Another advantage of the CRISPR-Cas system is that it allows multi-targeting (Ma et al., 2015). It has also been demonstrated that at least four guide DNA sequences can be included in the same construct (Lowder et al., 2015). Along with the gene stacking technologies, these new advances in genome editing capabilities will greatly reduce the number of transgenes and in the meantime allow for coordination of gene expression.

### Conclusion and Perspectives

Since the discovery of the C4 photosynthesis, there has been a keen interest to introduce this more efficient mechanism into C3 crops for increased productivity. Progress in C3-to-C4 engineering has been slow, however, due to a lack of understanding of the mechanisms underlying C4 photosynthesis. This status has changed recently with a number of important findings in C4 research (Furbank, 2017; Sedelnikova et al., 2018) and technical breakthroughs (Zhang et al., 2019; Lee et al., 2020; Zhu et al., 2020). Several new model plant species have also been introduced, such as *Setaria viridis* (a C4 monocot; Brutnell et al., 2010), *Cleome gynandra* (a C4 dicot; Newell et al., 2010), and the grass *Alloteropsis semialata* (Lundgren et al., 2016). Along with other well-established model species, such as rice (a C3 monocot) and *Arabidopsis thaliana* (a C3 dicot), these plants should allow us to define a core set of genes necessary for C4 photosynthesis as well as a C3 gene regulatory network on which C4 traits can be built. C3-to-C4 engineering is a complex project requiring collaborative efforts from the community. The C4 rice project is a good example (von Caemmerer et al., 2012). It can be envisioned that C4 rice will be made available in the near future, thus ushering in a new wave of Green Revolution.

## Author Contributions

HC conceived the project and wrote the manuscript.

## Funding

This work was supported by the Florida State University, Northwest Agriculture and Forest University and the National Science Foundation of China (grant no. 31871493).

## Conflict of Interest

The author declares that the research was conducted in the absence of any commercial or financial relationships that could be construed as a potential conflict of interest.

## Publisher’s Note

All claims expressed in this article are solely those of the authors and do not necessarily represent those of their affiliated organizations, or those of the publisher, the editors and the reviewers. Any product that may be evaluated in this article, or claim that may be made by its manufacturer, is not guaranteed or endorsed by the publisher.

## Footnotes

^1^ http://esa.un.org/unpd/wpp/index.htm
